# *Helicobacter pylori* Eradication Therapy in Nonulcer Dyspepsia is Beneficial

**DOI:** 10.4103/1319-3767.39629

**Published:** 2008-04

**Authors:** Mohammed Q. Khan

**Affiliations:** Section of Gastroenterology, Department of Medicine (MBC-46), KFSH and RC, Riyadh 11211, Saudi Arabia

Non-ulcer dyspepsia (NUD) is a common condition, and no therapy is dramatically effective in treating this disorder. It is therefore vital that there should be a reliable evidence for the efficacy of treatments prescribed to NUD patients. This updated systematic review suggests that H. pylori eradication has a small but statistically significant benefit in treating NUD.

One-half of the world's population has ***Helicobacter pylori*** (***H. pylori***) infection, with an estimated prevalence of 30% in North America[1–2] compared with a prevalence of 80–90% in the developing world.[3] The annual incidence of new ***H. pylori*** infections in industrialized countries is approximately 0.5 per 100 persons of the susceptible population compared with three or more per 100 persons in the developing countries.[4,5] In southern part of Saudi Arabia, ***H. pylori*** was present in 81% of nonulcer dyspepsia (NUD) patients (with normal esophago-gastroduodenoscopy [EGD]) reported[6] in 1993, but a recent report[7] after 11 years (2004) from the same area, showed a significant reduction (53%) in the incidence of ***H. Pylori.*** This reduction is most likely secondary to improved socioeconomic and hygienic conditions, in addition to widespread treatment of ***H. Pylori*** in dyspepsia patients. Risk factors for acquiring ***H. pylori*** infection include residence in a developing country, poor socioeconomic conditions, family overcrowding, and possibly an ethnic or genetic predisposition.

Dyspeptic symptoms affect 15–40% of the adult population in the western world.[8] Most affected individuals do not have structural or biochemical alterations that could explain these symptoms and, thus, are classified as having nonulcer or functional dyspepsia.[9] The pathophysiology of NUD is poorly understood; it may be associated with changes in motility, secretion, or sensitivity of the digestive tract.[10–12] Data from epidemiological studies are controversial in terms of the association of ***H. pylori*** with NUD symptoms.[13–15] Clinical studies focusing on the symptomatic benefits of eradicating ***H. pylori*** infections in patients with NUD have yielded conflicting results.[16,17] The majority of these studies were performed in low-prevalence ***H. pylori*** infection populations. [18–22] There are few studies reporting the consequences of eradicating these bacteria in infected functional dyspeptic patients in high-prevalence populations. Brazil is a country with a high ***H. pylori*** prevalence, with 60–87% of the adult population being infected with the bacteria.[23–25]

***H. pylori*** infection is the main cause of peptic ulcer and eradication therapy successfully cures this chronic relapsing and remitting disease.[26] This discovery has made an enormous difference to patients with peptic ulcer disease, but this accounts for only 5–10% of the population that has dyspepsia.[27] The majority of patients with dyspepsia have a normal endoscopy and in the absence of predominant reflux symptoms these patients are defined as having NUD.[28] The pathogenesis of this condition is uncertain and is likely to be multifactorial. ***H. pylori*** infection may have a role in this disorder, as the organism causes a chronic inflammatory response and has effects on gastric acid secretion.[29] The effect of ***H. pylori*** eradication therapy on NUD symptoms has been evaluated in several large, well designed, randomized controlled trials (RCTs).[30] Systematic reviews of RCTs can be a powerful method of assessing the effectiveness of a therapy. Unfortunately, many high-quality systematic reviews have been published with differing conclusions. This review aims to show that H. pylori eradication is beneficial and cost-effective in the treatment of NUD.

## DEFINITION OF NONULCER DYSPEPSIA

Dyspepsia is a common complaint in clinical practice; therefore, its management should be based on the best evidence. Dyspepsia has often been loosely defined; the most widely applied definition of dyspepsia is the Rome Working Teams formulation, namely chronic or recurrent pain or discomfort centered in the upper abdomen.[31] Predominant epigastric pain or discomfort helps to distinguish dyspepsia from gastro-esophageal reflux disease; in the latter the dominant complaint is typically heartburn or acid regurgitation, but there may be a distinct epigastric component that is confusing.[32] Frequent reflux symptoms (twice a week or more) probably impair quality of life and are generally considered to identify GERD until proven otherwise.[33–36] Clinical trials in dyspepsia have used various definitions and have often not distinguished obvious GERD from dyspepsia, making interpretation of treatment responses problematic.

## INITIAL EVALUATION OF THE PATIENT WITH DYSPEPSIA BY “TEST-AND-TREAT” STRATEGY

In the primary care clinic, the underlying pathology in patients with dyspepsia often is unknown. Rather than recommending endoscopy for all patients, most national guidelines suggest a “***test-and-treat***” strategy.[37–39] With this approach, patients who have symptoms of dyspepsia should be tested for ***H. pylori*** using a noninvasive method if they are younger than 45–55 years (depending on the guidelines) and do not have “red flags” for malignancy or complicated ulcer (e.g., dysphagia, early satiety, protracted vomiting, anorexia, loss of more than 10% of body weight, melena, rectal bleeding, abdominal mass, previous peptic ulcer disease, jaundice, and family history of gastric cancer). Several recent economic analyses show that the ***test-and-treat*** strategy improves symptoms and is cost-effective compared with other strategies.[40,41] A long-term follow-up study comparing a test-and-treat strategy vs. prompt endoscopy in patients with dyspepsia showed that the former reduced the number of endoscopies performed as well as the number of antisecretory medications administered.[42]

## EVIDENCE FOR TEST-AND-TREAT OR ACID SUPPRESSION

***H. pylori*** prevalence of <10% in the local community is the cutoff point for deciding to use empiric acid suppression rather than test-and-treat. The rationale for noninvasive ***H. pylori*** testing is the identification of underlying peptic ulcer disease. For example, in Scotland where the incidence of peptic ulcer is high, McColl ***et al.*** showed that in patients with dyspepsia and a positive C13 urea breath test had a duodenal ulcer (DU) in 40% and gastric ulcer (GU) in 13%; those who were breath test negative had a DU in 2% and GU in 3%.[43] Other studies suggest that between 20 and 60% of patients with dyspepsia who are ***H. pylori*** infected will have underlying peptic ulcer disease, but this varies widely depending upon the background incidence of peptic ulcer.[44,45] Cost-effectiveness studies in the United States suggest that when the prevalence of ***H. pylori*** infection in patients with functional dyspepsia is <12% or when the prevalence of ***H. pylori*** infection in patients with peptic ulcer disease is <48%, initial empirical treatment with a proton pump inhibitor is preferable.[46] Others have suggested that when ***H. pylori*** infection decreases below 20%, empiric PPI therapy starts to dominate test-and-treat in uninvestigated dyspepsia.[47]

## TEST-AND-TREAT *H. PYLORI* VS. PLACEBO IN DYSPEPSIA IN THE COMMUNITY

There are data indicating a small benefit for treating ***H. pylori*** empirically in those with the infection in the community (nonpatients). In a UK community trial, 32,929 individuals were invited and 8455 attended and were eligible; 2329 were positive for ***H. pylori*** and were assigned active treatment or placebo, with 1773 (76%) returning at 2 years.[48] There was an absolute risk reduction of 5% for upper GI symptoms on active therapy vs. placebo, although quality of life was unchanged. Presumably much of this benefit is explained by the treatment of undiagnosed peptic ulcer disease.

## TEST-AND-TREAT *H. PYLORI* VS. USUAL MANAGEMENT OF UNINVESTIGATED DYSPEPSIA IN PRIMARY CARE

Chiba ***et al.*** conducted a randomized placebo-controlled trial in 36 family practices in Canada; they randomized 294 ***H. pylori***-positive patients to omeprazole plus antibiotics or omeprazole plus placebo for 1 week and then arranged follow-up by family physicians for usual care.[49] They found eradication resulted in no or minimal symptoms in 50% of patients compared with 36% in the placebo-therapy arm at the end of 12 months. It is of interest that this benefit was observed despite including some GERD patients in this trial. The eradication therapy arm also reduced costs by Canadian $53 per patient. Allison ***et al.***[50] in a study in primary care in the United States observed no cost-benefit of test-and-treat over usual care although symptoms were significantly reduced in the test-and-treat arm.

## TEST-AND-TREAT *H. PYLORI* VS. PROMPT EGD IN PRIMARY AND SECONDARY CARE

There is consistent empiric evidence that a test-and-treat strategy is at least equivalent to prompt endoscopy in terms of outcomes. Lassen ***et al.*** randomized 500 patients (including older patients) in primary care with dyspepsia to either ***H. pylori*** test-and-treat or prompt endoscopy.[51] They found that there were no differences in symptomatic outcomes or quality of life between the groups at 1 year, although the endoscopy group had a slightly higher patient satisfaction score of questionable clinical significance. The authors also identified a reduction in the number of endoscopic procedures performed in the test-and-treat arm. Heaney ***et al.*** in Ireland evaluated dyspepsia patients <45-year-old referred to an open-access endoscopy unit who were ***H. pylori***-positive on noninvasive testing.[52] Patients here were randomized to either empiric ***H. pylori*** therapy or immediate EGD. They found that more patients became symptom free in the ***H. pylori*** eradication arm than in the prompt endoscopy arm. Jones ***et al.*** evaluated 232 patients in primary care, of whom 141 underwent testing and treatment for ***H. pylori***; 91 who had previously undergone endoscopy comprised the control group.[53] Although not a randomized controlled trial, they identified similar clinical outcomes but lower costs in the test-and-treat group at 1 year. Because this was a retrospective, unmatched nonconsecutive controlled study, the results are difficult to interpret. Additional randomized trial data[54] and a Cochrane metaanalysis[55] suggest overall that prompt EGD and test-and-treat have similar efficacy.

## *H. PYLORI* TESTING AND PATIENTS REASSURANCE

Other evidence supports the view that ***H. pylori*** testing may provide adequate patient reassurance. Patel ***et al.*** evaluated 193 dyspepsia patients under the age of 45 years.[56] Seventy of these patients were ***H. pylori***-seronegative without alarm features, 90 were seropositive for ***H. pylori***, and 23 had alarm features; the ***H. pylori***-positive patients and those with alarm features underwent prompt endoscopy. No difference in outcome or satisfaction was detected between the groups in follow-up after referral back to their primary care physician. The study concluded that a simple blood test provides similar reassurance to that of endoscopy and also results in reduced return visits and prescriptions.

## DOES *H. PYLORI* ERADICATION INCREASES THE RISK OF GERD?

It is controversial whether eradication of ***H. pylori*** infection increases the risk of development of reflux esophagitis or reflux symptoms.[57,58] However, it appears likely that this risk is only present in those with a predisposition to GERD who also have severe gastritis in the body or fundus that impairs acid secretion, which is reversed with ***H. pylori*** eradication; this is likely to be uncommon in most parts of the United States.[59] Hence, this issue while much discussed should not be a major clinical concern when contemplating test-and-treat, unless convincing data to the contrary arise. Progression of ***H. pylori*** gastritis may occur on acid suppression and some have suggested ***H. pylori*** eradication should be considered for all patients requiring long-term acid suppression, which seems reasonable.[60]

## *H. PYLORI* ERADICATION IN NUD PREVENT PEPTIC ULCER DISEASE

Eradicating ***H. pylori*** in patients with NUD may offer benefits beyond symptom improvement. Studies have reported that peptic ulcers develop in 1–14% of patients with NUD when followed over extended periods.[61–63] A placebo-controlled study from Taiwan found that ***H. pylori*** eradication reduced the 1-year incidence of peptic ulcer in patients with ulcer-like functional dyspepsia but not in those with dysmotility-like or unclassifiable NUD.[62] With these thoughts in mind, the decision of whether to test for and treat ***H. pylori*** in NUD should be individualized taking into consideration patient concerns as well as the presence of risk factors for peptic ulcer disease (age, NSAID^]^ use) and gastric malignancy (ethnic background, family history of gastric malignancy). It could be argued that ***H. pylori*** eradication therapy reduces symptoms in NUD because of the treatment of occult peptic ulcer disease that was not present at the index endoscopy. This may be the case; however, pragmatically the data still suggest that ***H. pylori***-infected patients with dyspepsia and a normal endoscopy will gain some benefit from ***H. pylori*** eradication therapy.

## RECENT META-ANALYSIS *H. PYLORI* AND NUD

At best, ***H. pylori*** eradication provides a small and highly variable symptomatic benefit in patients with NUD. Although a metaanalysis of 10 studies failed to demonstrate an improvement in symptoms with eradication therapy,[64] but an updated systematic review of 17 trials revealed a small but statistically significant benefit (NNT = 18).[65] The American College of Gastroenterology suggests an empiric trial of acid suppression with a proton pump inhibitor for 4–8 weeks as an option for initial treatment of dyspepsia in areas with a low prevalence of ***H. pylori*** infection.[66] The effect may be statistically significant, but the clinical significance of this finding is less clear. Moayyedi ***et al.*** constructed a health economics model that suggests ***H. pylori*** eradication is cost-effective in NUD,[67] although this was from a UK health care perspective and may not apply to other countries. Systematic reviews of RCTs are the best available tool for evaluating the effects of many health care interventions and methodology in this area is developing rapidly. Where systematic reviews disagree, as in this instance, the rigorous approach taken means that the work can be replicated and discrepancies understood. We have shown that it is important to regularly update systematic reviews in fields that are rapidly developing. The Cochrane Collaboration, through its policy of regular updates of all published reviews and electronic publication on The Cochrane Library[68] facilitates such an approach. An advantage of systematic review and meta-analysis methodology is that small treatment benefits can be detected that no individual trial has the power to detect. ***H. pylori*** eradication therapy seems to have just such an effect in improving the symptoms of NUD.

In summary, on the basis of these evidences, it is acceptable to offer ***H. pylori*** eradication therapy to the infected patients with functional dyspepsia. The results also imply that offering ***H. pylori*** eradication therapy empirically to those with otherwise uninvestigated dyspepsia who are infected is reasonable even if ulcer disease is unlikely. Moreover, ***H. pylori*** eradication in those with documented functional dyspepsia may help to prevent ulcer disease. The application of a test-and-treat strategy should be based on local prevalence of ***H. pylori*** infection. High prevalence of ***H. pylori*** among local populations in Saudi Arabia and recent migration of laborers and workers community from the developing countries made test-and-treat as the preferable nonendoscopic strategy. In low-prevalence populations (e.g., high-socioeconomic areas, where the background prevalence of ulcer or ***H. pylori*** infection is low), an alternative strategy is to prescribe first a course of antisecretory therapy empirically for 4–8 weeks. If the patient fails to respond or relapses rapidly on stopping antisecretory therapy, then the test-and-treat strategy is best applied before consideration of referral for EGD. EGD is not mandatory in those who remain symptomatic as the yield is low; the decision to perform endoscopy must be based on clinical judgment.
